# Medical management of a child with congenital generalized lipodystrophy accompanied with progressive myoclonic epilepsy: A case report

**DOI:** 10.1097/MD.0000000000018121

**Published:** 2019-11-27

**Authors:** Yi Zhang, Xiaofei Chen, Feixiang Luo, Lihua Jiang, Jialu Xu, Shuohui Chen

**Affiliations:** a Department of Neurology; b Department of Gastroenterology; c Neonatal Intensive Care Unit; d Department of Infectious Diseases, The Children's Hospital, Zhejiang University School Of Medicine, National Clinical Research Center For Child Health, Hangzhou, China.

**Keywords:** BSCL2 mutation, congenital generalized lipodystrophy, epilepsy enteral nutrition

## Abstract

**Rationale:**

Congenital generalized lipodystrophy (CGL) is a rare autosomal recessive hereditary disease. It is associated with metabolic complications and epilepsy is rare.

**Patient Concerns and Diagnoses:**

One child with BSCL2 mutation and CGL accompanied by progressive myoclonic epilepsy

Diagnosis: He was diagnosed with epilepsy, CGL, and severe malnutrition.

**Interventions:**

He was treated with sodium valproate, baclofen, aripiprazole, benzhexol, and lamotrigine for epilepsy.

**Outcomes:**

After 16 days of medical treatment for epilepsy, the disease was improved and the child was discharged with gastric tube inserted for the management of malnutrition.

**Lessons:**

CGL and progressive myoclonic epilepsy is rare, and the epilepsy is partially refractory to treatments. In this particular case, the nutritional status was compromised as a complication of progressive myoclonic epilepsy and had to be managed.

## 1 Introduction

Congenital generalized lipodystrophy (CGL) is a rare autosomal recessive hereditary disease, with an incidence of about 1/10,000,000.^[1]^ CGL is accompanied by a series of metabolic disorders such as hyperglycemia, hyperinsulinemia, insulin resistance, diabetes, and hypertriglyceridemia.^[2,3]^ CGL with the most sever phenotype is caused by BSCL2 mutation. Epilepsy and progressive myoclonic epilepsy can also be observed, but the co-occurrence of CGL and epilepsy is rare.^[3–5]^ The treatment of CGL itself is mainly based on the metabolic manifestations.^[6–9]^

Here, we report 1 child with BSCL2 mutation and CGL accompanied by progressive myoclonic epilepsy admitted at our hospital on November 27, 2017. After 16 days of medical treatment for epilepsy, the disease was improved and the child was discharged with gastric tube inserted for the management of malnutrition.

## 2 Case presentation

A boy of 9 years and 3 months old was hospitalized in 2011 for repeated convulsion for over 6 years. He was diagnosed with CGL by genetic diagnosis (BSCL2, homozygous mutation c.782.dupG; the parents were both with heterozygous mutation) in 2012. Seizure appeared again in 2014, but without urinary or fecal incontinence. Dynamic electroencephalogram suggested epileptiform discharges (with high possibility of generalized seizure), and the boy was diagnosed with epilepsy. The child received oral sodium valproate (0.5 g, tid) starting in February 2014, but the symptoms were not well controlled. He received oral lamotrigine (62.5 mg, qm; 75 mg, qn), but the child was found with increased symptoms. Aripiprazole (2.5 mg, qn) and benzhexol (1 mg, bid) were administered orally, and muscular tension improved. Impact therapy with high-dose methylprednisolone (0.4 g) was conducted in August 2014 and the previous drugs were resumed with prednisone acetate (10 mg, bid). Hand shaking and seizures were still found. Electroencephalogram suggested high levels of epileptiform discharges. The child was treated thrice with methylprednisolone impact therapy. Starting early 2017, the child's condition aggravated gradually. Muscular tension increased gradually. Baclofen (5 mg, tid) was added in September 2017. Then the seizures appeared 3 to 4 times per month.

Physical examinations at admission showed that the child was in poor mental state. Although the child appeared to be an adolescent, the subcutaneous fat all over the body had disappeared. A lipoma of 0.5 × 0.5 cm was found on the left upper eyelid. The extremities were with well-developed muscles. Abdominal wall varicosis was found, and the Babinski sign was negative. He was diagnosed with epilepsy, congenital CGL, and severe malnutrition.

In November 2017, muscular tension was elevated when awake, with occasional opisthotonus, treated with midazolam (0.5 μg/kg/minute). Sleeping blood pressure was 78/34 mm Hg, which increased to 108/50 mm Hg after intravenous injection of normal saline. The triglyceride levels were 0.78 mmol/L.

On the morrow, involuntary movements were still found, and the dose of midazolam was increased to 0.75 μg/kg/minute. Sleeping blood pressure was 79/36 mm Hg, and the blood glucose levels were 3.9 mmol/L. After intravenous injection of 5% glucose and sodium chloride injection (250 ml) + 10% potassium chloride injection (5 ml), blood pressure increased to 88/42 mm Hg, and blood glucose level to 4.2 mmol/L.

On the following day, the child was with poor appetite, with cough. A gastric tube was inserted and the boy received nasogastric feeding of milk (250 ml, Q6H). Midazolam was stopped, and clonazepam (1 mg, tid) was given. Chloral hydrate was administered for symptomatic treatment. As hypotension and hypoglycemia still appeared in the night, nasogastric feeding of milk was conducted beforehand.

Late November 2017, nasogastric feeding of peptamen junior milk (120 ml, Q4H) was conducted. Six times of large volumes of yellow watery stools were excreted within 24 hours. The skins around the anus was slightly red. Nasogastric feeding of montmorillonite powder (3 g, tid) and *Saccharomyces boulardii* (0.25 g, bid), and intravenous liquid infusion were conducted. Blood glucose was 3.7 mmol/L in the night, which increased to 5.7 mmol/L at 1 hour after feeding.

In December 2017, opisthotonus was evident. Sputum sound was noticed and sputum aspiration was conducted. Nasogastric feeding of 90 ml of peptamen junior milk and skim milk (75 ml, Q4H) was conducted. Muscular tension was slightly elevated, the child was with opisthotonus, and the extremities were twisting. Chloral hydrate was given and intravenous injection of ceftriaxone (1.5 g, qd) and ambroxol (15 mg, qd) was conducted. Nasogastric feeding of 120 ml peptamen junior milk and skim milk (100 ml, Q4H) was conducted.

**Figure 1:**
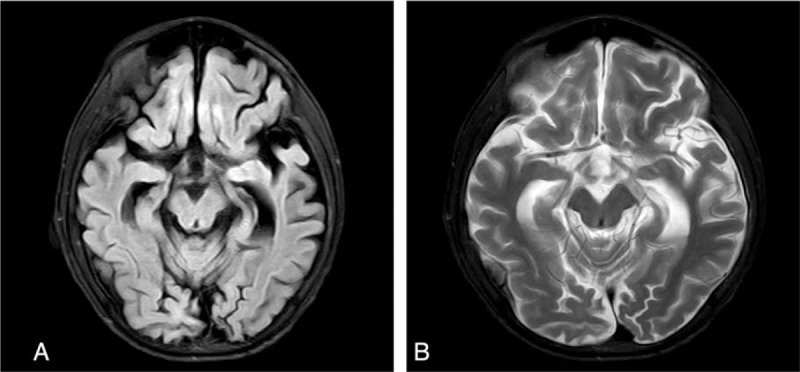
Cerebral magnetic resonance imaging (MRI) on November 28, 2017. Cerebral magnetic resonance imaging examinations on November 28, 2017 showed atrophic changes of the cerebrum accompanied by basal ganglia atrophy (A: FLAIR;B: T_2_WI).

From December 4 to 7, 2017, elevated muscular tension of the extremities and involuntary shaking of the body were still found. Nasogastric feeding of 10 ml chloral hydrate was provided. Cerebral magnetic resonance imaging (MRI) examinations showed atrophic changes of the cerebrum accompanied by basal ganglia atrophy (Fig. 1). Video electroencephalogram showed abnormal electroencephalogram, with large amounts of focal epileptiform discharges. The child was discharged on December 12, 2017 with symptoms.

On March 12, 2019, the child was conscious but with poor mental state. All subcutaneous fat had disappeared. Paroxysmal muscular tension elevation was evident when the child was awake. The child could not eat by himself, and was on nasogastric feeding. Peptamen junior milk and skim milk was provided, about 900 ml every day. Self-made fruit and vegetable juice was also provided, along with some enteral nutrient solution. The energy intake was 1750 kcal/d. Nasogastric feeding of sodium valproate (0.5 g, bid), lamotrigine (62.5 mg qm, 75 mg qn), trihexyphenidyl (1 mg, bid), clonazepam (reduced to 1 mg, bid, due to the excessive laryngeal secretions), and topamax (12.5 mg, bid) was conducted; aripiprazole was stopped. The child was hospitalized several times due to pulmonary infection, but the detailed treatments were unclear. The liver and renal functions were normal, and the blood glucose levels were also normal during follow-up.

## 3 Discussion

The boy had a confirmed homozygous mutation in the *BSCL2* gene and the typical physical characteristics of CGL (lack of adipose tissues and hypertrophy of the muscles of the extremities), but the blood triglyceride and glucose levels were low. This boy was unable to feed because of progressive myoclonic epilepsy. This undernutrition probably explained that the typical hypertriglyceridemia and hyperglycemia were absent.

Progressive myoclonic epilepsy mainly occurs in childhood or adolescence and accounts for about 1% of epilepsy cases.^[10]^ Various types of epileptic seizures, central nervous system dysfunctions such as cognitive impairment and ataxia can also be found in the patients.^[11]^ All those manifestations were observed in the case presented in this report. Opri et al suggested that CGL and progressive myoclonic epilepsy could segregate together, based on their observation of 3 cases with both diseases.^[3]^ They observed subcortical atrophy and T2 hyperintensities in the caudate and lenticular nuclei at MRI. In the present case, MRI showed atrophic changes of the cerebrum accompanied by basal ganglia atrophy.

The case reported here had the c.782dupG mutation in the BSCL2 gene. This mutation has been reported by Wu et al^[12]^ in 1 patient with compound heterozygous mutations in BSCL2 (c.782dupG and c.G565T) with CGL who developed general dystonia in adulthood, but without epilepsy or cognitive impairment. It is possible that progressive myoclonic epilepsy only develops in patients with homozygous c.782dupG mutation, but this will have to be verified.

Despite having been treated with so many antiepileptic drugs and sedatives, the liver and renal functions of the child were still normal, and no symptoms of urinary calculi were found. The neurological symptoms of the child were relatively severe, which were inconsistent with the previously reported cases.^[3–5]^ These findings underscored the importance of alimentary control. In addition, interventions could be applied for the child to reduce complications and improve the quality of life. With so many drugs being used for the treatment, management of the drugs is very important and it is crucial to obtain the parents’ cooperation. In addition, the physicians must be available to react quickly in case of sudden change in symptoms and they have to provide a close follow-up. Local community hospitals also play an important role in the management of the child. The parents had to be educated about the methods of changing the nasogastric tube, nasogastric feeding, extubation, and emergent handling of tube obstruction and asphyxiation, to ensure the quality of home enteral nutrition. Psychological care had to be provided for the parents to increase the anti-pressure ability, and thus provide better family support for the child. The parents of the child were both with good temper and got along with each other. The economic condition of the family was also good. After educations during the hospitalization, the home caring was sufficient, and the child was well cared at home.

## 4 Conclusion

In conclusion, CGL and progressive myoclonic epilepsy is rare, and the epilepsy is partially refractory to treatments and difficult to manage. In this particular case, the nutritional status was compromised as a complication of progressive myoclonic epilepsy and drugs taken to manage epilepsy, and had to be managed by intragastric feeding.

## Author contributions

**Conceptualization:** Yi Zhang.

**Data curation:** Yi Zhang.

**Formal analysis:** Yi Zhang.

**Funding acquisition:** Yi Zhang.

**Methodology:** Yi Zhang.

**Project administration:** Xiaofei Chen, Lihua Jiang.

**Supervision:** Feixiang Luo, Shuohui Chen.

**Validation:** Jialu Xu.

**Visualization:** Feixiang Luo, Lihua Jiang.

**Writing – original draft:** Yi Zhang.

**Writing – review & editing:** Yi Zhang,Shuohui Chen.

## Acknowledgment

The study was supported by the Zhejiang Traditional Chinese Medicine Science and Technology Plan(2020zb140).
